# Mechanisms of pathogenicity in the hypertrophic cardiomyopathy-associated TPM1 variant S215L

**DOI:** 10.1093/pnasnexus/pgad011

**Published:** 2023-01-21

**Authors:** Saiti S Halder, Michael J Rynkiewicz, Jenette G Creso, Lorenzo R Sewanan, Lindsey Howland, Jeffrey R Moore, William Lehman, Stuart G Campbell

**Affiliations:** Department of Biomedical Engineering, Yale University, New Haven, CT 06511; Department of Physiology/Biophysics, Boston University, Boston, MA 02215; Department of Biomedical Engineering, Yale University, New Haven, CT 06511; Department of Biomedical Engineering, Yale University, New Haven, CT 06511; Department of Internal Medicine, Columbia University, New York, NY 10032; Department of Biological Sciences, University of Massachusetts Lowell, MA 01854; Department of Biological Sciences, University of Massachusetts Lowell, MA 01854; Department of Physiology/Biophysics, Boston University, Boston, MA 02215; Department of Biomedical Engineering, Yale University, New Haven, CT 06511

**Keywords:** hypertrophic cardiomyopathy, tropomyosin, engineered heart tissue

## Abstract

Hypertrophic cardiomyopathy (HCM) is an inherited disorder often caused by mutations to sarcomeric genes. Many different HCM-associated TPM1 mutations have been identified but they vary in their degrees of severity, prevalence, and rate of disease progression. The pathogenicity of many TPM1 variants detected in the clinical population remains unknown. Our objective was to employ a computational modeling pipeline to assess pathogenicity of one such variant of unknown significance, TPM1 S215L, and validate predictions using experimental methods. Molecular dynamic simulations of tropomyosin on actin suggest that the S215L significantly destabilizes the blocked regulatory state while increasing flexibility of the tropomyosin chain. These changes were quantitatively represented in a Markov model of thin-filament activation to infer the impacts of S215L on myofilament function. Simulations of in vitro motility and isometric twitch force predicted that the mutation would increase Ca^2+^ sensitivity and twitch force while slowing twitch relaxation. In vitro motility experiments with thin filaments containing TPM1 S215L revealed higher Ca^2+^ sensitivity compared with wild type. Three-dimensional genetically engineered heart tissues expressing TPM1 S215L exhibited hypercontractility, upregulation of hypertrophic gene markers, and diastolic dysfunction. These data form a mechanistic description of TPM1 S215L pathogenicity that starts with disruption of the mechanical and regulatory properties of tropomyosin, leading thereafter to hypercontractility and finally induction of a hypertrophic phenotype. These simulations and experiments support the classification of S215L as a pathogenic mutation and support the hypothesis that an inability to adequately inhibit actomyosin interactions is the mechanism whereby thin-filament mutations cause HCM.

**Significance Statement**

For families threatened by inherited cardiomyopathies, screening to determine individual risk relies heavily on the identification of reliable genetic markers. To speed this process, we created a computational pipeline to mechanistically predict effects of uncategorized TPM1 variants. As a proof-of-concept, we studied the TPM1 variant S215L, using molecular dynamic simulations and scaling techniques to predict its physiological behavior. These predictions showed good agreement when compared with in vitro data acquired using purified proteins and engineered heart tissues. They also provide a detailed description of how S215L might induce structural and functional changes that lead to disease. The results suggest that our multiscale pipeline has future potential as a tool for assessing the pathogenicity of novel HCM-causing mutations and subsequent risk-stratification.

## Introduction

Hypertrophic cardiomyopathy (HCM) is a myocardial disease that is characterized by thickening of the left ventricular wall, hypercontractility, and microstructural tissue abnormalities such as interstitial fibrosis and myocyte disarray. HCM affects 1 in 500 people and is most often caused by autosomal dominant sarcomeric gene mutations (1). HCM is also the leading cause of sudden cardiac death among young adults (2–4). This, along with other heart-related complications such as heart failure, stroke, and blood clots among HCM patient populations can be prevented if accurate risk stratification is available (5). At present, risk stratification relies on finding and annotating pathogenic mutations in HCM probands. Developing a well-understood genotype–phenotype correlation of different HCM variants can better inform risk-stratification strategies.

Over 1,000 HCM-causing sarcomeric mutations have been identified, most of which correspond to thick filament-encoding genes such as myosin heavy chain 7 (MYH7) and MYBPC3 (6). However, according to Coppini et al., mutations to thin-filament proteins (actin, troponin, and tropomyosin) lead to increased likelihood of LV dysfunction and heart failure in adult patients (7). Because mutations in thin-filament proteins are often associated with severe phenotypes, they can provide useful information about fundamental disease mechanisms.

Among all variants of thin-filament proteins, 16% occur in α-tropomyosin (TPM1) (8). Tropomyosin is a key regulator of muscle contraction and has a two-stranded alpha-helical coiled-coil structure. The alpha-helices consist of continuous seven residue (a–g) repeats (known as heptads) that are characteristic of coiled-coil proteins (9). Tropomyosin also has a modular pseudo-repeating pattern of 39 residues which allow binding of the coiled coil to successive actin subunits. Each of these pseudo-repeats is further divided into alpha-zones (that are closer to actin subunits 1 and 3) and beta-zones (that are closer to actin subunits 2 and 4) (10).

There are no particular structural locations within tropomyosin that can be specifically associated with HCM-causing mutations. HCM, dilated cardiomyopathy and left ventricular noncompaction-associated point mutations have been identified across various heptad positions, in different pseudo-repeats, and in both the alpha and beta bands of tropomyosin (11). This indicates that structural position alone is a poor predictor of variant phenotype or severity. Furthermore, many of these variants are unique to specific families such that the feasibility of linkage analyses is limited, and they are seldom performed. Because of these factors, the pathogenicity of newly discovered TPM1 variants is extremely difficult to assess. Efficient and accurate means of characterizing TPM1 variants would better inform risk-stratification strategies and increase the potential for early intervention and prevention of adverse outcomes.

Previous work characterizing TPM1 variants has used various techniques to shed light on the structural and functional changes that occur in tropomyosin. This includes molecular dynamic (MD) simulations, computational models, atomic force and electron microscopy, circular dichroism, and differential scanning calorimetry (12–15). Tropomyosin regulatory function has been assessed biochemically using in vitro motility (IVM) assays, actomyosin ATPase assays, and cosedimentation assays (16–18). Despite the different techniques employed, the structure–function relationship of many TPM1 variants remains incompletely understood.

In this study, we aimed to characterize HCM-associated TPM1 variant S215L through a unified multiscale assessment that included atomistic simulations, computational thin-filament modeling, regulated IVM assays, and finally human engineered heart tissues (EHTs). By integrating the results of these diverse methods, we have produced a succinct and consistent description of the molecular consequences of TPM1 S215L and how they result in contractile abnormalities. We further demonstrate that hypercontractility at the cell level activates hypertrophic gene expression pathways. These data strongly support the classification of TPM1 S215L as a HCM-causing mutation and illustrate the potential for integrative multiscale investigation to efficiently determine TPM1 variant pathogenicity.

## Results

### Differences in altered atomic level interactions

**Fig. 1.**
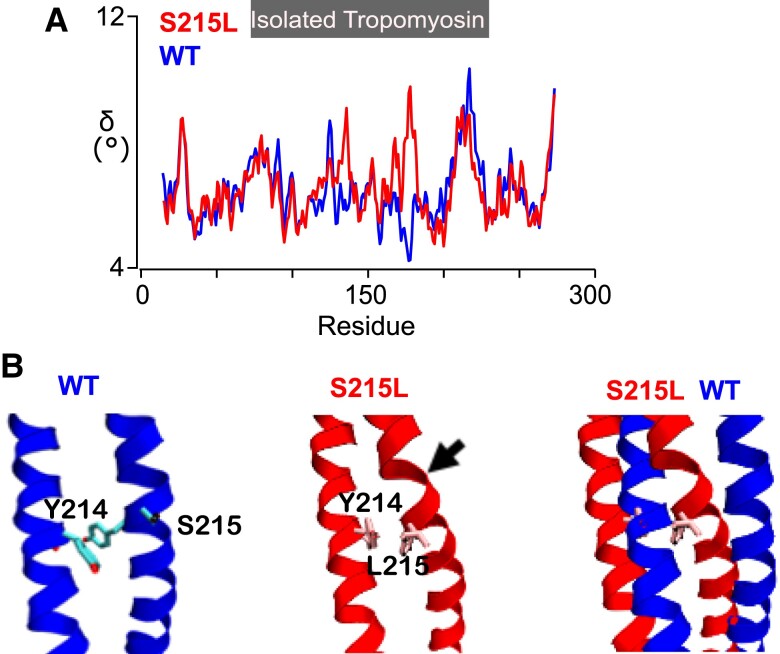
Molecular dynamics simulations. A) A comparison of mutation-induced local changes in flexibility (δ) of isolated tropomyosin (TPM1). B) A detailed view of the isolated TPM1 structure: WT (left), S215L (center), and overlay (right). Arrow indicates the position where a bend in the TPM1 chain is observed.

We performed atomistic simulations in order to investigate potential molecular impacts of a missense Serine to Leucine substitution at the 215th residue of TPM1. Using MD simulations, we were able to estimate the changes in local flexibility along the length of a “free-floating” tropomyosin molecule (Fig. 1A) which revealed that there are some differences in the atomic level interactions. In the wild-type (WT) structure (Fig. 1B, left), S215 is solvent exposed. MD simulations show that mutation to Leu215 (Fig. 1B, center) packs the leucine side chain in a hydrophobic interaction with Y214, a residue that makes up the core of the molecule, resulting in local distortions of the tropomyosin coiled coil (arrow). The bend in the tropomyosin chain can be observed in the overlay of WT and S215L (Fig. 1B, right).

The distortions we observe in Fig. 1B could propagate throughout the tropomyosin molecule and alter its regulatory activity. In order to study potential impacts of TPM1 S215L on tropomyosin regulatory behavior, interaction energies between tropomyosin and actin were also extracted from MD simulations. Analysis of the tropomyosin interaction energies with actin (Table 1) shows that the interaction energies of the S215L substitution are less favorable than WT when tropomyosin is placed in the blocked (B) regulatory state. In the closed (C) regulatory state, S215L leads to more favorable tropomyosin–actin interaction energies. Overall, these changes suggest slight destabilization of the B state and stabilization of the C state by S215L.

**Table 1.** Tropomyosin–actin–troponin I interaction energies and tropomyosin–actin interaction energies in the B and C states, respectively, from the molecular dynamics simulations.

Genotype	B-state (kcal/mol)	C-state (kcal/mol)	*K*_BC_
WT	−5,108.7 ± 280.9	−3,895.4 ± 236.9	0.76
S215L	−4,581.3 ± 232.9	−4,426.2 ± 206.2	0.97

### Lowered stiffness and B-state destabilization predict increased Ca sensitivity and contractility

**Fig. 2.**
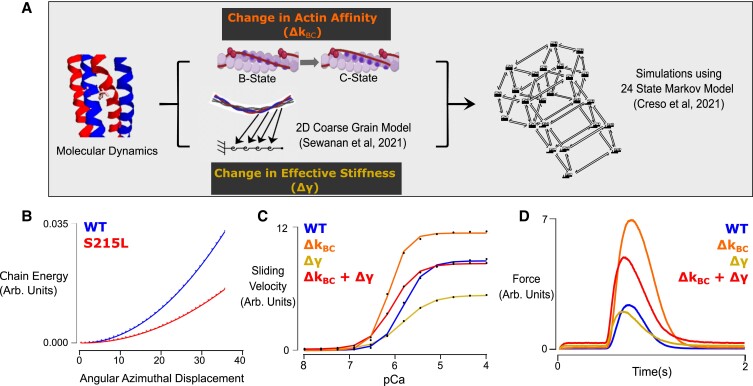
Computational modeling. A) A schematic explaining which parameter changes were considered and how they were calculated. B) Chain energy vs. azimuthal displacement showing decreased effective stiffness of S215L calculated using the 2D coarse-grain model. (C) Steady-state simulation and (D) Isometric twitch simulation using 24-state Markov model.

We next sought a prediction of how the putative molecular-scale consequences of S215L might impact physiological function at the sarcomere level. This was accomplished by inputting MD data into a coarse-grain model of tropomyosin mechanics and then into a Markov model of thin-filament regulation (Fig. 2A). Local changes in tropomyosin chain angular displacements (Fig. 1B) were used as parameters in the coarse-grain model to estimate the effective tropomyosin chain stiffness. The chain energy calculated over a range of azimuthal displacements (Fig. 2B) demonstrated a 54% drop in tropomyosin stiffness when S215L is introduced (compared with WT). The blocked-closed equilibrium constant, *K*_BC_, was calculated using the proportional change in Gibbs free energy informed by changes in actin–tropomyosin interaction energies in the B and C state obtained from molecular modeling (Table 1). The change in effective stiffness γ and the increased *K*_BC_ suggested by MD-based calculations of actin–tropomyosin interaction energy were then used as mutation-dependent parameters for a 24-state Markov model of thin-filament function. This model was used to predict the effects of the S215L mutation on the Ca^2+^-regulated IVM assay and isometric twitch force. Simulations were repeated while implementing each of the MD-predicted mutation effects on their own or the two in combination (Fig. 2C). In steady-state sliding velocity simulations, an isolated change in γ resulted in a considerable decrease in maximal activation while keeping pCa_50_ relatively the same compared with WT. On the other hand, a change in *K*_BC_ only resulted in a large leftward shift in pCa_50_ along with an increase in maximal activation. The combined effect of the two is a leftward shift in pCa_50_ with only a slight decrease in maximal activation. In a similar fashion, the isometric twitch predictions (Fig. 2D) indicated a slightly smaller peak force with a faster twitch for a 54% decrease in γ. Meanwhile, the presumed S215L-caused increase in *K*_BC_ resulted in a nearly three-fold increase in peak force with an elongated twitch. The combined effect of both mutation-based parameter changes was a two-fold increase in peak force with a 17% faster time to peak, a 40% slower relaxation time and a substantial increase in baseline force.

### Increase in calcium sensitivity and increased end–end bond strength

**Fig. 3.**
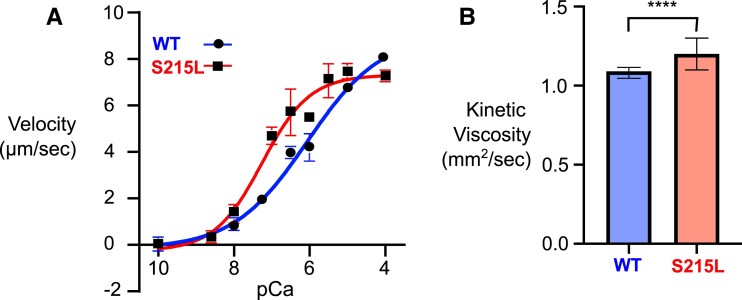
In vitro assays. A) In vitro motility assay showing increase in Ca sensitivity for S215L. B) Increase in kinetic viscosity in S215L.

After having predicted how the calcium-dependent cooperative activation could be changing in S215L, we sought to validate the observations through experimentation. Thin filaments reconstituted with mutant S215L TPM show significantly increased calcium sensitivity for actomyosin interactions via a shift of 1.0 pCa_50_ units (Fig. 3A). The pCa_50_ values and Hill coefficients are summarized in Table 2. Furthermore, we observed a significantly higher viscosity in S215L compared with WT which suggested an increase in end–end bond strength in the mutant (Fig. 3B).

**Table 2.** In vitro motility assay parameters.

	WT	S215L
pCa50	6.06 ± 0.29	7.25 ± 0.19
Hill coefficient	0.44	0.69

### S215L EHTs demonstrate slowly relaxing hypercontractile twitches compared with WT

**Fig. 4.**
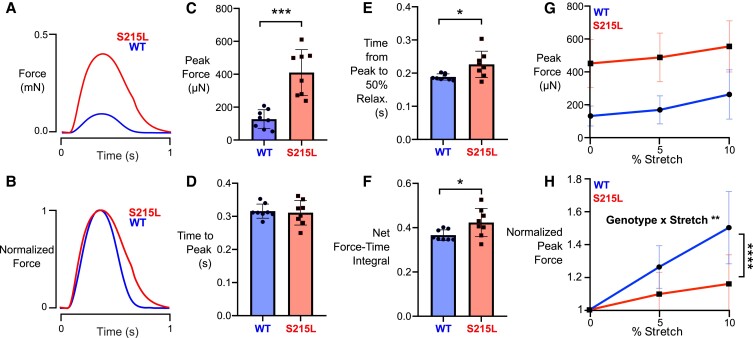
Basic twitch properties of WT and S215L EHTs while pacing at 1 Hz. A, B) Sample force traces. C) Peak force. D) Time from start of stimulus to peak force. E) Time from peak to 50% relaxation. F) Net force time integral. G) Length-dependent activation of EHTs showing peak forces at 0, 5, and 10% stretch. No statistical test performed. H) Length-dependent activation of EHTs showing normalized peak forces (data from each EHT normalized to its own peak force at culture length, i.e. 0% stretch). Curves significantly different by two-way ANOVA, significant interaction between stretch and genotype (*P* = 0.0069). Result of post-hoc pairwise comparison indicated on the graph.

We next aimed to find out how active contractile properties were being affected due to the TPM1 S215L mutation. We utilized two isogenic cell iPSC cell lines: one for WT and one with a CRISPR/Cas9-induced homozygous TPM1 S215L mutation. EHTs seeded with iPSC-derived WT or S215L cardiomyocytes were cultured for 14 days and then subjected to contractile testing (Fig. 4). The isometric twitch profiles of S215L and WT EHTs were markedly different (Fig. 4A and B). We found that at culture length while being paced at 1 Hz, S215L EHTs showed a dramatic three-fold increase in the strength of isometric contraction compared with WT (Fig. 4C). While there was no significant difference in the time it took the EHTs to reach the peak force (Fig. 4D), mutant EHTs took a significantly longer time to relax, with the relaxation time increasing by 20% compared with WT (Fig. 4E). The slowly relaxing hypercontractile twitch led to a nearly 16% increase in the calculated normalized force-time integral (measured as the area under the force-time traces) compared with WT (Fig. 4F). We also measured the length-dependent activation of the EHTs by applying a 0, 5, and 10% stretch and measuring the corresponding peak twitch forces. The peak forces at each stretch level were normalized to that seen at a 0% stretch in the same tissue. We observed that while tissues from both groups increased peak contractile force at increasing stretch levels (Fig. 4G), EHTs expressing TPM1 S215L were relatively less sensitive to stretch (Fig. 4H).

### S215L EHTs demonstrate increased diastolic stiffness that can be attributed mainly to diastolic actin-myosin cross-bridge activity

**Fig. 5.**
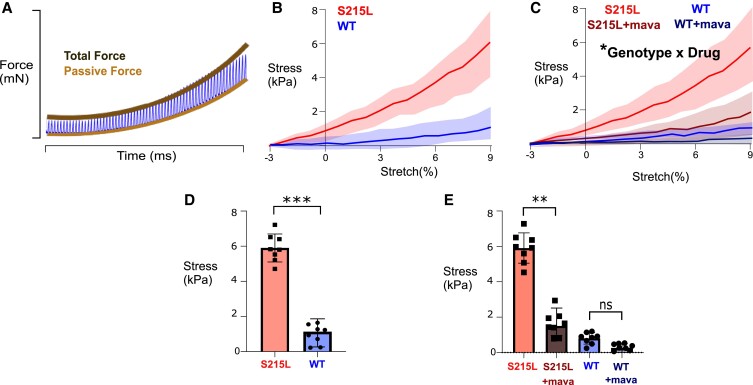
Detailed analysis of diastolic forces in WT and mutant EHTs. A) A schematic showing the sample force trace as the stretch is applied. B) Passive stress in WT and S215L EHTs (*N* = 8, curves significantly different by two-way ANOVA). C) Passive stress in WT and S215L EHTs followed by 30 min of 2 µM mavacamten treatment [*N* = 8, curves significantly different by three-way ANOVA; significant interaction of genotype × drug (*P* = 0.0167)]. D) Stress values at 9% stretch showing results for Tukey post hoc of graph shown in B. E) Stress values at 9% stretch showing results for Tukey post hoc of graph shown in C. The shaded areas in B and C represent the standard deviation of individual tissue data.

In accordance with model predictions of elevated diastolic force due to TPM1 S215L (Fig. 2D), we examined the diastolic behavior of EHTs undergoing ramp stretches. EHTs were slowly stretched from a slack length (−3% relative to culture length) to a stretch of 9%, all while being electrically paced at 1 Hz. The diastolic force in this case is defined as the minimum force level observed between stimulus events. The force trace from this protocol can be seen in Fig. 5A. Diastolic force was extracted from post-hoc analysis of raw traces and diastolic stress was obtained by dividing raw force by EHT cross-sectional area. Force transducer output was zeroed at slack length (−3% stretch) to provide a common baseline across tissues. This analysis ultimately yielded diastolic stress–stretch curves (Fig. 5B). Two-way ANOVA showed an overall significant interaction between stretch and TPM1 genotype in affecting diastolic stress (Fig. 5B). A post-hoc analysis of these data revealed that the TPM1 S215L tissues dramatically increased diastolic stress at 9% stretch compared with WT (Fig. 5D).

We hypothesized that the increased diastolic stress we observe in Fig. 5B could be attributed to the presence of residual actin–myosin cross-bridges during the diastolic interval. To investigate whether this was true, we performed another similar experiment where we recorded the diastolic stress of WT and mutant EHTs before and after treatment with 2 µM mavacamten for 30 min. The diastolic stress traces (Fig. 5C) showed that treatment with mavacamten, a potent small molecule myosin inhibitor, was able to reduce the diastolic stress in both groups. It was observed that after cross-bridge inhibition, S215L continues to maintain an increased diastolic stress compared with WT. However, the relative drop in diastolic stress after an acute mavacamten treatment is higher in S215L than in WT, resulting in a significant genotype–drug interaction (*P* = 0.0167). The post-hoc analysis of these data is illustrated in Fig. 5E.

### No differences observed in calcium handling

**Fig. 6.**
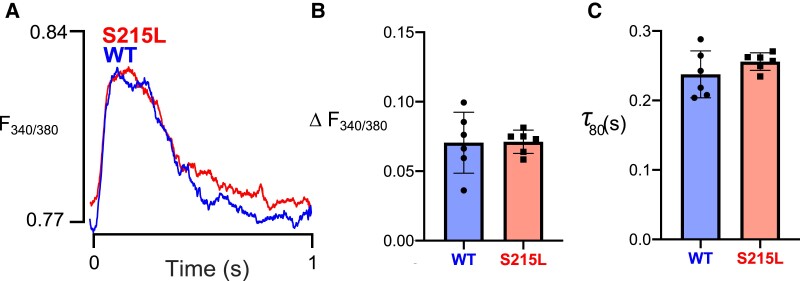
A) Sample calcium transient observed in the EHTs. Calcium handling properties: (B) change in F340/F380 ratio and (C) τ_80_ (time decay constant calculated after fitting an exponential decay curve starting from 80% of maximal value).

We next assessed intracellular calcium transients in Fura-2 loaded EHTs to determine whether differences in calcium handling could explain the hypercontractility observed in TPM1 S215L EHTs. Fig. 6A shows two sample calcium transient traces observed for WT and S215L EHTs. From these transients, we computed the maximal change in the ratio of Fura-2 absorbances at 340 and 380 nm excitation wavelengths, Δ*F*_340/380_. We also calculated the time constant of calcium transient decay (starting from 80% of the maximal value), denoted at τ_80_. Both WT and S215L EHTs exhibited similar values for both parameters, and no significant difference was observed using unpaired Student's t test.

### S215L iPSC cells show increased hypertrophy at the cellular level compared with WT

**Fig. 7.**
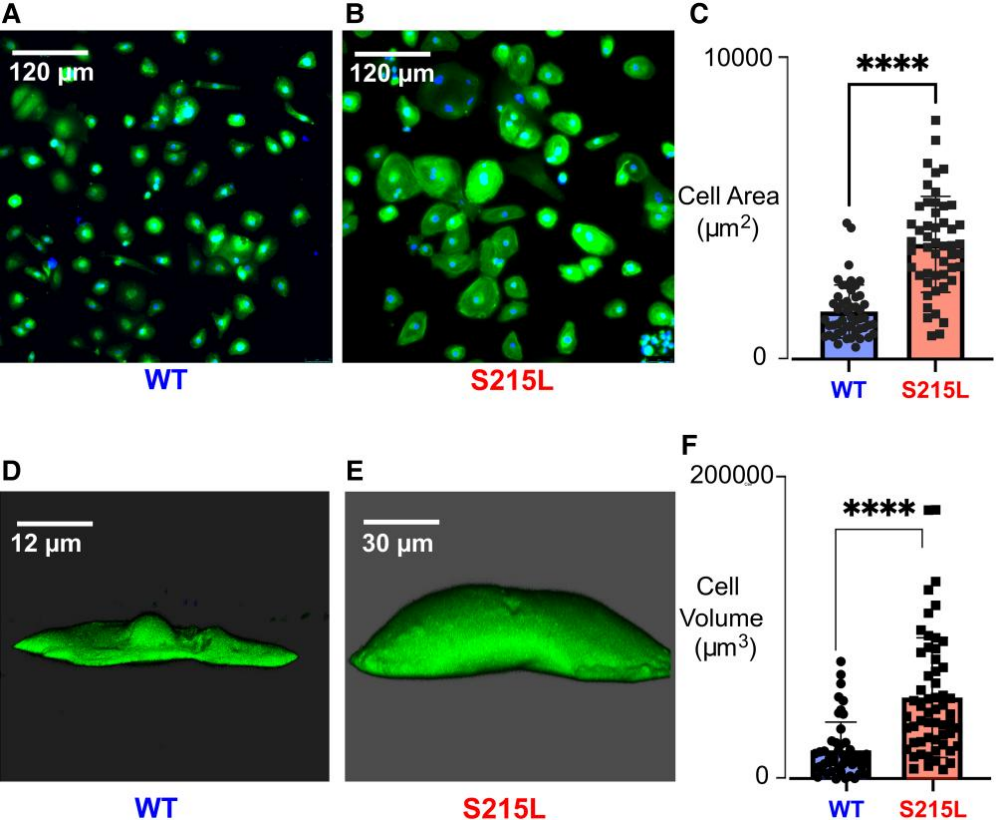
Cell size comparison in 2D and 3D. Immunofluorescent staining of cardiac troponin T (green) and DAPI (blue) on a monolayer of iPSC-CM cells (A) WT and (B) S215L. (C) Cell area measurement for 50 cells. 3D reconstruction of CellTracker Live Imaging (green) of (D) WT and (E) S215L cells combining Z-stacks captured using a laser confocal microscope. (F) Cell volume measurement for 50 cells.

The mechanical characterization of S215L EHTs was qualitatively consistent with predictions made through mathematical modeling, namely that this mutation would increase twitch force and elevate the resting or diastolic force. However, the EHTs showed a much more dramatic increase in both systolic and diastolic forces compared with simulation outputs. We hypothesized that the apparently exaggerated response in tissues could be due to cardiomyocyte hypertrophy occurring in consequence of the S215L mutation even in the short-time interval over which EHTs are cultured. To test this hypothesis, we first examined the size of TPM1 S215L-expressing cardiomyocytes in 2D culture. Cells in a 2D monolayer were fixed and immunostained for cTnT. The images of WT and S215L cells are shown in Fig. 7A and B, respectively. The area for each cell was calculated using ImageJ. It was observed that at the cellular level, differentiated S215L cardiomyocytes increased in cell size compared with WT cells (Fig. 7C). To investigate whether this increase in cell area was due to a greater degree of cell spreading or an increase in overall cell volume, we performed live imaging on a monolayer of WT and S215L cells. 3D reconstruction of WT and S215L cells showed a visible increase in cell volume (Fig. 7D and E). Cell volumes, computed using an image analysis software (Imaris by Oxford Instruments), showed a >three-fold increase in S215L cells compared with WT.

### S215L EHTs demonstrate upregulated hypertrophic gene markers

**Fig. 8.**
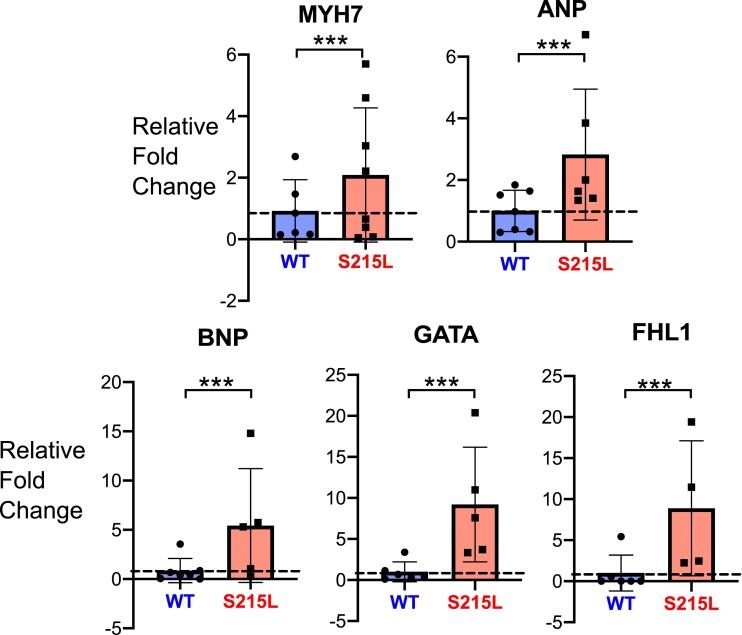
Hypertrophic gene expression profile of EHTs showing relative fold change for: myosin heavy chain 7 (MYH7), atrial natriuretic peptide (ANP), brain natriuretic peptide (BNP), GATA-binding protein 4 (GATA4), four-and-a-half LIM domain protein-1 (FHL1).

To further test the hypothesis that S215L is promoting cardiomyocyte hypertrophy, we homogenized EHTs and isolated RNA for analysis. RT-qPCR analysis of transcripts from the lysed mutant EHTs showed that there was a greater than two-fold increase in expression of MYH7 and atrial natriuretic peptide (ANP) transcripts compared with WT. Mutant EHTs also expressed five-fold more brain natriuretic peptide (BNP), GATA, and four-and-a-half LIM domain protein-1 (FHL-1) transcripts (Fig. 8). For all five genes, the observed expression differences were significant.

### A heterozygous mutation in S215L results in an intermediate phenotype

**Fig. 9.**
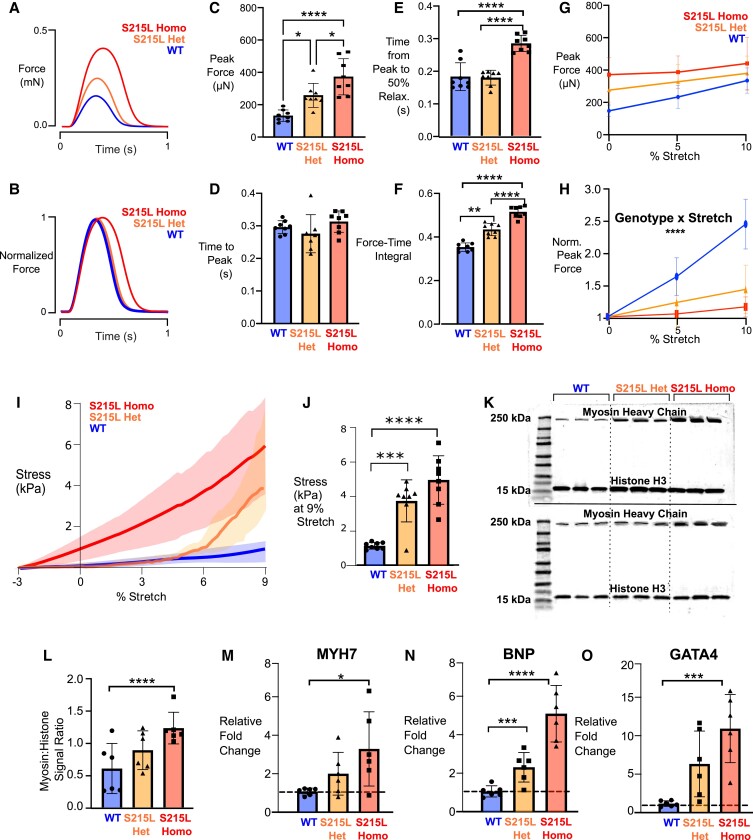
Comparison of wild type (WT), S215L heterozygous (S215L het), and S215L homozygous (S215L homo) EHTs. A and B) Sample force traces. C) Peak force. D) Time from start of stimulus to peak force. E) Time from peak to 50% relaxation. F) Net force time integral. G) Length-dependent activation of EHTs showing peak forces at 0, 5, and 10% stretch. No statistical test performed. H) Length-dependent activation of EHTs showing normalized peak forces (data from each EHT normalized to its own peak force at culture length i.e. 0% stretch). Curves significantly different by two-way ANOVA, significant interaction between stretch and genotype (*P* < 0.0001). Result of post-hoc pairwise comparison indicated on the graph. I) Passive stress in WT, S215L het, and S215L homo EHTs (*N* = 8, curves significantly different by two-way ANOVA). The shaded areas represent the standard deviation of individual tissue data. J) Stress values at 9% stretch showing results for Tukey post hoc of graph shown in I. K) Two western blots showing the myosin heavy chain and histone H3 bands. L) The myosin/histone signal ratios calculated from the two blots shown in I. M–O) Hypertrophic gene expression profile of EHTs showing relative fold change for: myosin heavy chain 7 (MYH7), brain natriuretic peptide (BNP) and GATA-binding protein 4 (GATA4).

We next aimed to find out how a more physiologically relevant heterozygous S215L mutation might impact the contractile properties of EHTs. To that end, we utilized another cell line containing a CRISPR/Cas9-induced heterozygous TPM1 S215L mutation that was isogenic to the two previous cell lines. Contractile measurements revealed that EHTs with heterozygous S215L mutation exhibited stronger isometric twitches than WT EHTs, but weaker isometric twitches compared with EHTs with homozygous S215L mutation (Fig. 9A and B). The peak forces generated in heterozygous S215L EHTs were 1.6 times higher compared with WT and 1.4 times lower compared with homozygous S215L EHTs (Fig. 9C). Although there were again no differences observed in the time taken by EHTs to reach peak force (Fig. 9D), homozygous S215L EHTs exhibited significantly delayed relaxation compared with both WT and heterozygous S215L EHTs (Fig. 9E). As a result of these differences, the calculated force-time integral of the heterozygous S215L EHTs also followed a similar intermediate trend as the peak force (Fig. 9F). We wondered if a heterozygous S215L mutation would impact the length-dependent activation of EHTs and obtained isometric twitch recordings at 0, 5, and 10% stretches. We observed that although there were positive force-length responses recorded for EHTs in all three groups (Fig. 9G), both mutant groups exhibited significantly flattened length-dependent activation compared with WT (Fig. 9H). Upon assessment of the diastolic stiffness displayed by these EHTs in response to stretch, it was observed that both mutant groups had increased stiffness compared with WT (Fig. 9I). Interestingly, the heterozygous S215L EHTs exhibited a decrease in average compliance with increasing stretch. Compared with WT EHTs, the passive stresses recorded at 9% stretch were 2.7 and 3.3 times higher in heterozygous S215L EHTs and homozygous S215L EHTs, respectively.

Given the evidence of hypertrophy in S215L mutant cells obtained previously, we wanted to investigate whether there were alterations in the myofibrillar content in heterozygous S215L cells as well. To answer that, we performed western blots with homogenized cell samples from each cell line. We stained the blots for myosin heavy chain and Histone H3 (Fig. 9K). The normalized ratios of the band intensities revealed that homozygous S215L cells demonstrated 2-fold higher myosin content while heterozygous S215L cells demonstrated 1.5-fold higher myosin content compared with WT cells (Fig. 9L). RT-qPCR analysis of transcripts from the lysed mutant EHTs showed that there was a significant increase in expression of MYH7, BNP, and GATA4 transcripts in homozygous S215L EHTs compared with WT (Fig. 9M–O). Heterozygous S215L EHTs demonstrated a statistically significant increase in BNP transcripts compared with WT.

## Discussion

Serine 215 in TPM1 is an amino acid residue that has been shown to be highly conserved in mammalian evolution (19). The amino acids serine and leucine are physiochemically very different. It is therefore understandable that a mutation at this residue results in a marked structural change. The mutation S215L was first identified in 2012 in children under 15 in South Indian populations (20). A study that used next generation sequencing to predict pathogenicity of HCM variants predicted TPM1 S215L to be a pathogenic variant with a variant frequency of 0.43 (21). In recent years, there have been several reported clinical instances of this variant. In 2016, TPM1 S215L was noted as one of multiple sarcomeric mutations observed in compound heterozygous HCM patients (22). In another study, S215L was identified in 1 of 1,535 patients in a cross-sectional study of HCM (23). Although S215L accounts for a small portion of all HCM-causing tropomyosin variants, there is clinical evidence that it results in an aggressive HCM phenotype when presented in a compound heterozygous context (24).

In this study, we undertook a thorough biomechanical analysis of the HCM-associated TPM1 variant S215L and traced its consequences across multiple spatial scales. MD simulations suggested significant S215L-induced changes in tropomyosin stiffness and actin interactions. Data from these simulations were used as a quantitative basis for changing two key parameters in a model of thin-filament activation, specifically decreasing the effective chain stiffness (*γ*) and increasing the blocked-closed equilibrium constant (*K*_BC_). This allowed prediction of how S215L might affect higher order behavior such as Ca^2+^-regulated thin-filament motility and isometric twitch contractions of intact cardiomyocytes. We predicted steady-state and twitch behavior by incorporating a change in *γ*, *K*_BC_, or both. Comparison of the model and experimental data showed that changing both parameters most closely recapitulated experimental behavior. The model and experimental data were in good qualitative agreement with regards to the effects of S215L, specifically the increase in myofilament Ca^2+^ sensitivity, hypercontractile twitch, and prolonged twitch relaxation time. We also characterized the effects of a S215L mutation in a heterozygous context and demonstrated that a single TPM1 S215L allele alone is also sufficient to cause alterations in transcription and protein levels that can lead to dysfunctional contractile phenotypes.

The increased calcium sensitivity, hypercontractility and altered actin–tropomyosin–troponin interactions also agreed with increased viscosity of TPM-S215L compared with WT. Viscosity measurements provide a measure of the end–end bond strength between adjacent tropomyosin molecules along the tropomyosin strand (25). The role of head-to-tail overlap region on tropomyosin interactions with actin has been previously established (26, 27).

Increases in calcium sensitivity and hypercontractility are hallmarks of HCM and have been previously reported in contractile evaluations of many HCM-causing mutations (17, 28–32). We noticed, however, that the hypercontractile behavior observed in our mutant EHTs was relatively exaggerated compared with the model prediction. By measuring calcium transients in our EHTs, we were able to rule out discrepancies in calcium cycling as a potential mediator of this unexpected increase in contractility. Instead, we wondered if the mutant iPSC-CMs could be exhibiting hypertrophic growth that ultimately manifested as enhanced EHT contractility. Indeed, there was a greater than two-fold increase in cell area, a three-fold increase in cell volume, and a two-fold increase in myosin content in S215L cells compared with WT. This suggests that the greater force produced by S215L EHTs was in part due to increased myofibrillar content. We observed no significant changes in the cross-sectional areas of the EHTs (Fig. S1). Interestingly, although both homozygous and heterozygous S215L EHTs were consistently hypercontractile across a range of varying preloads, they fared significantly worse than WT EHTs in terms of relative length-dependent activation. We wondered whether this was a result of an increase in resting sarcomere length. However, histological measurement of sarcomere length in fixed EHTs showed no significant differences in the distance between alpha-actinin bands (Fig. S2). Dampening of length-dependent activation has previously been observed in HCM patient-derived cardiomyocytes harboring the M281T TPM1 missense mutation (33).

Changes in contractility caused by TPM1 S215L do not appear to be the consequence of altered calcium handling. Calcium mishandling has been implicated as a common mechanism in some experimental studies of HCM (34, 35) but not in others. Riaz et al. showed more rapid SR calcium uptake in MYH7 R723C mutant cardiomyocytes, which would tend to compensate for slowed detatchment rate of mutant myosin (31). Sewanan et al. showed that there were no observable differences in calcium handling between EHTs seeded using TPM1 E192K patient-derived cells and a WT control (29). Whatever the ultimate role of calcium handling changes in the progression of HCM, our results at least suggest that that they are not essential for producing the most proximate disease phenotypes in the case of tropomyosin mutations.

In addition to the systolic abnormalities, we also observed evidence of diastolic dysfunction in S215L EHTs. The first hint that the mutation could exhibit disrupted diastolic function was the abnormalities in the actin–tropomyosin interaction energies which indicated a destabilization of the blocked state of tropomyosin. This alteration stabilizes the closed state and destabilizes the blocked state of tropomyosin which lowers the energy barrier to convert between states for thin-filament activation at physiologically relevant calcium levels. Increased activation of the thin filament was predicted in the model to produce hypercontractility and prolonged relaxation time and was confirmed with our homozygous S215L EHTs. Abnormalities in muscle relaxation can be indicative of underlying dysfunction. One previous study shows that there is a strong correlation between extent of left ventricular hypertrophy and regional left ventricular relaxation abnormalities (36). In fact, isovolumetric relaxation time is clinically used as a noninvasive yet reliable method to detect diastolic dysfunction among HCM patients (37). We do note, however, that a similar increase in relaxation times was not observed with heterozygous S215L EHTs.

We also observed substantially increased baseline force in our isometric twitch simulations. Upon measuring the diastolic stress in our EHTs, we discovered that mutant homozygous EHTs exhibited greater diastolic stress compared with WT. We hypothesized that presence of calcium-independent residual cross bridges may be contributing to the increased diastolic stress. We subsequently incubated our tissues with high-dose mavacamten which would inhibit cross-bridge formation and allow us to measure the passive stress. We observed that mavacamten incubation reduced the stress in both mutant and WT EHTs. Statistical tests revealed that the loss of diastolic stress after myosin inhibition is more pronounced in S215L than it is in the WT, indicating that there are more residual cross-bridges present in S215L than in WT. Previously, TPM1 mutants V95A, D175N, and E180G were shown to result in an increase in the number of active cross-bridge cycles at pCa 8, resulting in an increased diastolic tension (38). A study of E192K EHTs from patient-derived cell lines by Sewanan et al. showed similar attenuation of cross-bridge-based stiffness upon treatment with mavacamten (29). This suggests that residual cross-bridge activity may be a common characteristic of HCM-causing TPM1 variants. Interestingly, in the case of heterozygous S215L EHTs, the passive stiffness resembled that of WT at low stretches but rapidly increased at higher stretches to resemble that of homozygous S215L EHTs. This behavior may have been due to more myosin heads switching from a super relaxed state at lower stretches to disorder relaxed state at higher stretches and binding with the mutant tropomyosin, thus resulting in diastolic dysfunction at higher stretches. This may also explain why an increase in relaxation time was not observed with heterozygous S215L EHTs during isometric twitch recorded at culture length. The effect of the heterozygous mutation on diastolic properties might only become prominent at higher preloads.

Upon comparing the passive stress under mavacamten treatment, we also observed that there is a persistent increase in stiffness in S215L compared with WT. This could be due to fibroblast-mediated remodeling or a change in titin-based stiffness. In response to systolic dysfunction, cardiac fibroblasts may be activated via multiple pathways to induce myocardial fibrosis (39). Myocardial fibrosis can lead to increased passive myocardial stiffness by collagen accumulation, collagen phenotype shift, and enhanced collagen cross-linking (40). Another in-vitro study showed increased titin-based passive stiffness in cardiomyocytes from failing human hearts with HCM (41).

We also sought molecular evidence that hypertrophic signaling pathways were active in homozygous S215L mutant EHTs. We tested our EHTs for biomarkers that are typically upregulated in HCM using RT-qPCR. Data show consistent upregulation of HCM markers MYH7, FHL1, ANP, and BNP. MYH7 and FHL1 were previously shown to be differentially expressed in HCM hearts (42, 43). Assessment of transcripts from heterozygous EHTs also showed a significant increase in BNP compared with WT. Ventricular expressions of ANP and BNP have been reported to be augmented in patients with HCM, obstruction, or diastolic dysfunction (44, 45). We also observed upregulation of GATA4 which is a critical transcription factor responsible for cardiogenesis. It was recently shown that suppression of GATA4 via Kindlin-2 can prevent cardiomyocyte hypertrophy (46). These data demonstrate that key HCM signaling pathways are activated in our mutant EHTs.

The only in vitro characterization of TPM1 S215L to date was by Gupte et al., who performed cosedimentation and actomyosin ATPase assays on purified protein (18). They showed that S215L results in a nearly two-fold decrease in tropomyosin-binding affinity for actin with hypersensitivity to calcium. These observations are consistent with our IVM experiments. Our IVM data also showed increased kinetic viscosity which indicates and increase in end–end bond strength. This may explain the observed increased cooperativity between the tropomyosin molecules, as evidenced by an increase in Hill coefficient of velocity–pCa curves. The increase in end–end bond strength may also be an indication that the S215L mutation results in long range structural changes in the protein away from the site of the mutation. All these deviations suggest that the S215L mutation results in major structural and functional changes in the tropomyosin protein which manifests as a pathological HCM-causing phenotype.

Our multiscale analysis and evaluation of the mechanistic changes caused by mutation S215L is not without some limitations. For the steady-state simulation, we set parameters of the WT to previous WT values reported in literature (14). However, the Hill coefficient of the IVM experiment for the WT was markedly different from previously reported Hill coefficients for control groups, although a similar shift in pCa50 in the model and in the experimental group was observed. There are several aspects that contribute to lower cooperativity in IVM experiments. pCa50 and cooperativity have been demonstrated to vary depending on the amount of myosin on the surface (47). The IVMA experiments presented were performed at the same surface loading and thus can be directly compared with reveal relative changes in calcium sensitivity for mutant vs. WT. Another factor that can contribute to differences under IVM assay conditions and intact sarcomeres can be related to thick filament-based effects. Since IVM is performed on myosin that has been applied to the cover glass surface in high-salt buffer, myosin will be in monomeric vs. filamentous form. Therefore, any myosin-conformation-based effects on the number of heads functionally available will not be assessed. In the presented experimental data, the mutation is in the thin filament thus we rely on comparing mutant with WT under the same conditions to reveal the effects of the S215L mutation in thin-filament activation specifically. Our integrated approach is then used to link these simplified experimental systems with higher order sarcomeric interactions in EHTs.

Another limitation of this work is that our MD simulations were not solved using explicit water. Using explicit water, while more accurate, would be more computationally expensive and time consuming. Instead, we chose to use implicit solvation by using an artificial frictional drag to stabilize the tropomyosin molecule using CHARMM 27 which is better suited for implicit calculations (48). Similar methods have been previously used and validated by other studies (29, 49–51). While we recognize that this is an approximation, it is useful for predicting relative changes between mutant and WT since both filaments were subjected to the same simulation conditions. The computational model also shows a mildly decreased time to reach peak force which is not observed in the isometric twitch for EHT. This may be a result of the model overemphasizing the rate of change of force production given the strong evidence of B-state destabilization seen in MD simulations.

**Fig. 10.**
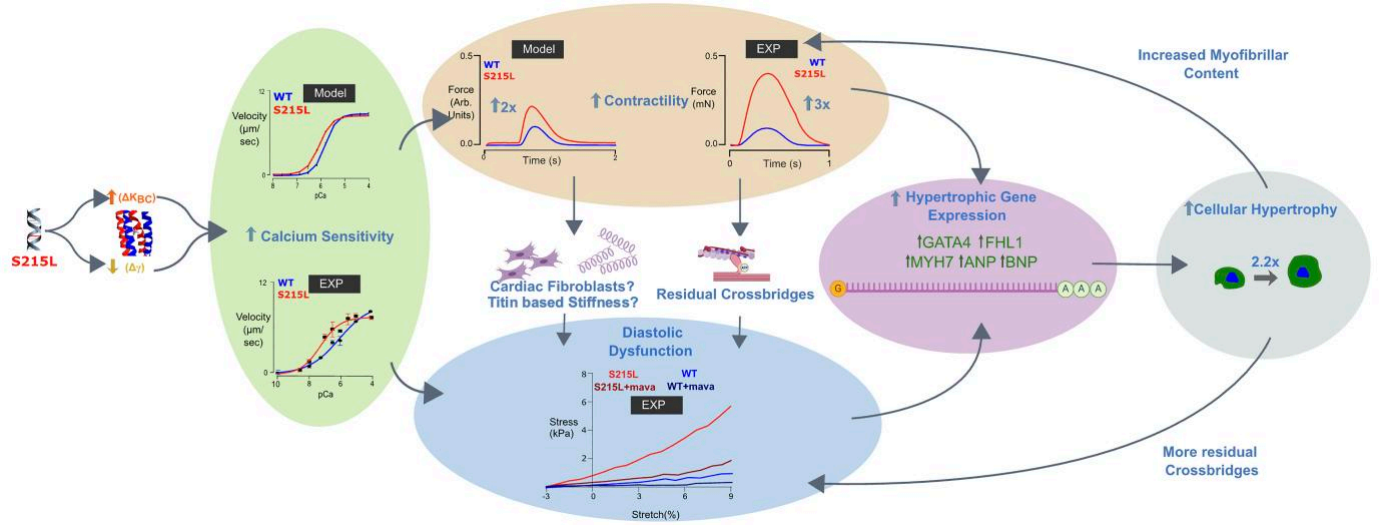
A schematic depicting the probable pathological mechanism in S215L.

This study joins others in a long-standing effort to translate atomistic predictions of thin-filament structural mutations into cardiac contractile phenotypes (29, 52, 53). Fig. 10 attempts to articulate an unbroken chain of cause-and-effect linking the genetic lesion TPM1 S215L to the induction of hypertrophic gene pathways. The degree to which MD simulations of S215L tropomyosin behavior succeeded in predicting appropriate muscle contraction changes suggests that goal of in silico genotype–phenotype predictions could ultimately be fulfilled. Future work will test the ability of the multiscale pipeline to assess the physiological consequences and severity of other novel TPM1 mutations, including those with modes of pathogenicity distinct from TPM1 S215L.

## Methods

### Molecular dynamics simulations

MD simulations of isolated tropomyosin dimers were performed as previously described (54). Briefly, the starting model was an ideal coiled-coil backbone where the tropomyosin side chains had been built to match crystal structure coordinates (55, 56). After mutations were incorporated, the structure was minimized in CHARMM using the CHARMM 27 force field and the Generalized Born with a simple SWitching (GBSW) implicit solvation model. Simulations were then run for 30 ns at 300 K with a frictional drag of 1/ps applied to solvent-exposed heavy atoms, a time step of 2 fs, and the SHAKE algorithm. Calculation of the local fluctuation of the superhelical backbone (*δ* angle) was calculated in CHARMM as described (54).

Models of the low- and high-calcium configurations of tropomyosin bound to actin were built starting from the coordinates of the modified cryoelectron microscopy structures as previously published (57–59). Periodic boundary conditions were used to generate neighboring actin/tropomyosin/troponin molecules along the thin filament aligned to the *z*-axis, thus simulating an infinite filament in both directions. In the starting model, there are no full tropomyosin chains (the chains are comprised of residues 1–81 or 12–284), so these were also extended before simulation. To build the full model, the coordinates were copied by applying actin symmetry after centering the model on the origin and aligning along the *z*-axis to place the neighboring molecules above and below the model along the filament axis. Then, the tropomyosin models were ligated together at places where the peptide bonds were closest to extend the tropomyosin chains to a full 284 residues. After this was done for both low- and high-calcium models, a combined model was created that had the low-calcium configuration of tropomyosin, troponin T residues 89–151, and troponin I residues 138–210 along one long-pitch side of actin and the high-calcium configuration of tropomyosin and TnT residues 89–151 on along the other long-pitch actin helix. Additional chains at the ends of the filament model were included as place fillers to account for space occupied by periodic copies during the addition of waters. Waters were then added to the system using the solvate plugin in VMD (60). The extra 4 actin, 8 tropomyosin, and 2 troponin T chains were then removed at this point, and the system was then ionized in VMD to 0.15 M NaCl and 3 mM magnesium chloride. The final system consists of 28 actin monomers, 8 tropomyosin chains, 4 troponin T chains, and 2 troponin I chains.

The interaction energies of WT and mutant tropomyosin in the C state were calculated as the sum of the Coulombic and Van der Waals energies between all the tropomyosin and all the actin atoms with a distance cutoff of 16 Angstroms. For the B state, the interaction energies were calculated in a similar manner first for all tropomyosin–actin atoms before adding in all tropomyosin–troponin I interactions. The final values were calculated as average and standard deviations from 500 MD frames over the last 10 ns of simulation.

### Computational modeling

Using the local changes in tropomyosin flexibility and a coarse-grained model previously described in Sewanan et al. (29, 61), we calculated the effective stiffness of the mutant TPM1 chain. Briefly, each half tropomyosin molecule was modeled as a flexible cable of different torsional springs each with their own torsional stiffness (the relative stiffness at each amino acid residue as calculated by MD). The model calculated the potential energy of the chain as one end was fixed and the other was moved through a range (0 to 35°) of θ, the azimuthal position of tropomyosin on actin. The calculated energies were fit to obtain a global effective tropomyosin chain stiffness γ.

From the interaction energies from the atomistic simulations in Table 1, it can be observed that WT tropomyosin–actin–troponin I interaction energy in the B state was much more negative than the WT tropomyosin–actin interaction energy C states. We predicted that this would result in a change in the blocked-closed equilibrium constant *K*_BC_.

We decided to use a 24-state Markov model previously described by Creso and Campbell to predict changes in steady state and isometric twitch conditions (62). Parameters of the control (WT TPM1) simulation were determined by picking the set of values that yielded matching Hill coefficients and pCa_50_ values previously published for thin filaments reconstituted with WT TPM1 as well as matching contractile properties for previously published isometric twitches (14). Given these constraints, the blocked-closed equilibrium constant *K*_BC_ was set to a value of 0.76 for the WT simulations. We solved for Gibbs free energy for the WT Δ*G*_WT_ using the Gibbs relation:


$$K_{\mathrm{BC},\mathrm{WT}}=e^{-\Delta G_{\mathrm{WT}}/\mathrm{RT}}$$

Using data from the MD simulations (Table 1), it was calculated that the interaction energy difference between B and C states was +1,213 kcal/mol for the WT, while it was only +155.1 kcal/mol in the mutant case. This meant that there was an 87.2% decrease in the magnitude of the blocked-closed interaction energy difference for the mutant compared with WT. Using this percentage, we calculated a proportional change in Gibbs free energy for the mutant, Δ*G*_mutant_. This value was plugged back into the Gibbs relation to obtain a *K*_BC_ for the mutant (*K*_BC, mutant_ = 0.97).

The changes in γ and *K*_BC_ were applied to the 24-state Markov model of the thin filament which was used to simulate both steady-state and isometric twitch conditions using parameters described in Table 3. Briefly, the model simulates the behavior of 26 regulatory units in series, tracking for each unit the states of tropomyosin and key domains of troponin C and troponin I. The simulations use either a steady-state Ca^2+^ concentration or a transient to elicit contractile activity over a set time interval. Force was output as the number of regulatory units in a myosin-bound state.

**Table 3.** Parameters used in the computational model.

Parameter	Set 1
$k_{\mathrm{Ca}}^{+}\;(\mu M^{-1}s^{-1})$	350
$k_{\mathrm{Ca}}^{-}(s^{-1})$	1,000
$k_{\mathrm{SP}}^{+}(s^{-1})$	180
$k_{\mathrm{SP}}^{-}(s^{-1})$	300
$k_{\mathrm{IP}}^{+}(s^{-1})$	620
$k_{\mathrm{IP}}^{-}(s^{-1})$	225
$k_{\mathrm{MD}}^{+}(s^{-1})$	550
$k_{\mathrm{MD}}^{-}(s^{-1})$	225
$k_{\mathrm{ref}}^{\mathrm{BC}}(s^{-1})$	675
*K*_BC_	0.76, 0.97
*f*_XY_(s^−1^)	225
*δ*	0.48
*λ*	0.008
*η*	9
*μ*	9
*γ*(mol^−1^kJ)	31.6, 60

To simulate IVM assay (IVMA) data, steady-state force values at various Ca^2+^ concentrations were obtained by simulating a 5-s interval to ensure attainment of steady-state conditions. The steady-state force was determined by averaging force over a window of the final 25% of each simulation. To convert these force values into sliding filament velocity, a simple proportionality (effective filament viscosity) between force and velocity was assumed (29). Steady-state force values at different Ca^2+^ concentrations were used to produce velocity–pCa plots which were fit using the Hill equation.

Twitch events were simulated by allowing the system to reach steady state at a diastolic Ca^2+^ concentration of 0.1 μM. The Ca^2+^ concentration was then allowed to produce a transient by increasing up to 1 μM based on data from Stull et al. (63).

Model scripting and data post-processing was conducted in MATLAB, while the Markov chain-Monte Carlo algorithm was implemented in CUDA C++ for parallel processing. Simulations were executed on an Nvidia GeForce RTX 2080Ti graphics processing card.

To ensure convergence of the stochastic model, the simulation time course was repeated 1,920 times on the GPU and the average force at each time step was calculated. To further reduce stochastic noise, twitch simulations were run 10 times and averaged to calculate twitch properties (diastolic force, peak force, time to peak force, and time to 50% relaxation).

## In vitro motility assays

Both WT and mutant S215L were expressed in *Escherichia coli* as described previously (64). After expression, tropomyosin was purified from cell extracts via three rounds of isoelectric precipitation and two rounds of ammonium sulfate cuts at 55 and 70%, respectively. Purified protein was ultimately dialyzed into storage buffer (55 mM KCl, 25 mM BES, 4 mM MgCl_2_, 5 mM EGTA, 1 mM DTT, pH 7.4) and stored at −20°C. Protein purity was confirmed via SDS-PAGE.

IVM assay was conducted in a nitrocellulose-coated flow chamber as described previously (Sundar et al., 2020). Briefly, chicken skeletal muscle myosin, diluted to 50 μg/mL in myosin buffer (300 mM KCl, 25 mM BES, 5 mM EGTA, 4 mM MgCl_2_, pH 7.4, 0.1 M DTT) was added to a nitrocellulose-coated flow chamber and incubated for 1 min. Once incubated, the unbound surface was blocked with 1 mg/mL BSA in myosin buffer by adding 25 μL to the flow cell and incubating for 1 min. Unbound protein was removed by washing the chamber with 25 μL of actin buffer (55 mM KCl, 25 mM BES, 50 EGTA, 4 mM MgCl_2_, pH 7.4, 0.1 M DTT). Unlabeled actin was added to the flow cell and incubated for 2 min to allow the actin to bind to the surface-bound catalytically inactive myosin heads, unbound actin was removed with an actin buffer wash of 25 μL actin buffer with 1 mM ATP followed by ATP removal with four washes in actin buffer without added ATP. TRITC-phalloidin labeled F-actin with 800 nM tropomyosin and troponin was added to the flow cell and incubated for 5 min. Movement was assessed after the addition of different concentrations of Ca^2+^ with motility buffer (40 mM KCl, 25 mM BES, 5 mM EGTA, 4 mM MgCl_2_, pH 7.4) in the presence of 1 mM ATP and oxygen scavengers (45 mg/mL catalase, 10 mg/mL glucose oxidase, and 40 mg/mL dextrose/glucose). Four to six videos at each pCa of the motile thin filaments were recorded and later analyzed using the MTrckJ ImageJ plugin. All experiments were repeated as triplicates and the values were averaged to determine filament velocity and fraction of filaments motile.

Myosin was purified from chicken pectoralis muscle obtained from freshly slaughtered chicken using established methods (65). Porcine cardiac troponin and actin were prepared from acetone powder as described previously (66, 67). Briefly, actin was stored for up to 6 months at 4°C in the presence of 1 mM NaN_3_ while troponin aliquots were flash frozen in liquid nitrogen and subsequently stored at −80°C until use.

### Viscosity measurements

Viscosity measurements were made using a Cannon-Manning Semi-Micro Viscometer as described previously (64, 68). For these experiments, mutant and WT tropomyosin were dialyzed into low salt buffer (10 mM imidazole, 2 mM DTT, 10 mM NaCl). All measurements for WT and mutant tropomyosin were measured in triplicate at 22°C).

### iPSC maintenance and differentiation

Isogenic cell lines were created by the University of Connecticut Health Sciences Human Genome Editing Core, using CRISPR/Cas9 to introduce the TPM1 S215L variant into a commercially available WT human induced pluripotent stem cell (iPSC) line (GM23338, Coriell Institute). Upon delivery, the iPSCs were differentiated into ventricular cardiomyocytes (69). First, iPSCs were plated on a Matrigel (Corning, 1:60 diluted) coated well for culture for 3–4 days in mTeSR medium (Stemcell Technologies, 05850) till ∼90% confluency and treated with 20 μM CHIR99021(Selleckchem) on day 0 for 24 h and 5 μM IWP4 (Stemgent) on day 3 in RPMI (Gibco)/B27 (Gibco) minus insulin media for 48 h. During the cardiac differentiation, media were changed every other day with RPMI/B27 minus insulin media. After beating, cardiomyocytes were cultured in regular RPMI/B27 (including insulin). Cardiomyocytes at day 14 were treated with 4 mM lactate (Sigma) in glucose-free medium for 4 days to obtain enriched CMs.

### Engineered heart tissue fabrication and active mechanical measurements

EHTs were made by iPSC-CMs into decellularized porcine myocardial slices according to our previously published protocol (70, 71). Briefly, 150 µm thick slices were obtained from porcine left ventricular free-wall blocks, mounted onto custom tissue culture cassettes and decellularized. The scaffolds were then treated with 10% FBS and 2% antibiotic-antimycotic (Thermo Fisher) overnight. iPSC-CMs were dissociated using TrypLE Express 1x (Thermo Fisher) followed by washing with PBS and manual pipetting. For this study, we seeded WT or S215L cell suspensions containing 1 million cells onto each scaffold and allowed them to incubate overnight. The EHTs were subsequently grown in DMEM + 2% B27 plus insulin for 14 days. After that, EHTs were assessed for their active contraction mechanics using a custom-built setup that utilizes a World Precision Instruments (WPI KG7) force transducer. The EHTs were kept in Tyrode's solution at 36°C and 7.3 physiological pH as they were paced at 1 Hz and stretched from culture length (0% stretch) to up to 10%. The resulting isometric twitch was analyzed in MATLAB to calculate peak force, time from stimulus to peak contraction, time from peak to 50% relaxation, and normalized tension-time integral. In order to characterize intracellular calcium dynamics, some EHTs were loaded with the ratiometric fluorescent indicator Fura-2 AM (Millipore) by incubation at room temperature for 20 min in loading solution (Tyrode's solution with 17 μg/mL Fura 2-AM, 0.2% Pluronic F127, and 0.5% Cremophor EL) and subsequently imaged at 36°C using a photometric system as previously described (70).

### Passive mechanical measurements and acute drug treatment

For passive mechanical measurements, EHTs were preconditioned by stretching them repeatedly, from slack length (−3% stretch) to a maximum stretch of 9% for three cycles at a rate of 0.015 mm/s (0.25% muscle length/s). After preconditioning, EHTs were paced at 1 Hz and the force was recorded as they were stretched from −3 to 9%. The diastolic force produced was extracted by a custom MATLAB script as previously described (71). To assess the effect of acute mavacamten treatment on EHTs, passive diastolic force was measured prior to addition of mavacamten. Tissues were incubated at 36°C in 2 μM mavacamten in Tyrode's for 30 min while being paced at 1 Hz. Passive diastolic force was measured again after incubation once the active force had equilibrated.

Optical coherence tomography (OCT) scans were performed on tissues to capture the cross-sectional area which was then calculated using ImageJ. The cross-sectional area was used to normalize the passive force measured and therefore calculate the passive stress of the tissues.

### qPCR of gene expression

Tissues were flash frozen after 14 days of culture. RNA was extracted using Trizol (Life Technologies) phase separation technique. After phase separation, RNA was precipitated using isopropyl alcohol. The resulting RNA pellet was resuspended in 100 μL of nuclease free water. The mRNA content was quantified using Nanodrop software. After this, a DNase I kit (Fisher Scientific) was used to perform a DNase treatment. iScript-cDNA synthesis kit (Life Science Research) was used to reverse transcribe mRNA to cDNA according to manufacturer's instructions. Quantitative PCR amplifications were performed using an IQ SYBR Green Supermix (Bio-Rad) with a total reaction volume of 15 μL containing 1 μL of cDNA, 1.5 μL of primers, and 5 μL of distilled water on a CFX96 Real-Time System (Bio-Rad) under the condition of 95°C for 3 min followed by 46 amplification cycles (95°C for 10 s, 58°C for 10 s, 72°C for 30 s). Glyceraldehyde 3-phosphate dehydrogenase was used as a reference gene for this experiment. Three biological replicates were performed for expression analysis of each gene. Primers used are listed in Table 4. All gene expression-fold changes are expressed relative to WT.

**Table 4.** Primers used in the study.

Primer	Description	Product size (bp)	Forward (5′–3′)	Reverse (5′–3′)
GAPDH	Glyceraldehyde 3-phosphate dehydrogenase	117	GAAGGTGAAGGTCGGAGTCA	TTGAGGTCAATGAAGGGGTC
ANP	Natriuretic peptide A	120	CAGGATGGACAGGATTGGAG	ACAGGAGCCTCTTGCAGTCT
BNP	Natriuretic peptide B	109	TTTGGGAGGAAGATGGACC	TGTGGAATCAGAAGCAGGTG
MYH7	Myosin heavy chain 7	271	ACACCCTGACTAAGGCCAA	AGCTTCTTCTGCAGCTGGC
FHL1	Four and a half LIM domains 1	105	ATCCCCACGCAGCACCTT	CACCTTGTAGCTGGAGGGAC
GATA4	GATA-binding protein 4	127	CCCGACACCCCAATCTC	CAGGCGTTGCACAGATAGTG

### Cell size measurements

Differentiated cardiomyocytes at day 18 were passaged at a low concentration onto Matrigel coated 12-well tissue culture plates. The cells were cultured in RPMI/B27 (including insulin) for 4 days after which they were fixed using 4% paraformaldehyde overnight at 4°C before immunostaining. The primary cardiac TnT antibody used was MS-295 p0-(Thermo Fisher) at a dilution of 1:500. The secondary antibody used was goat anti-mouse IgG, Alexa Fluor 488 (Thermo Fisher, A-11029) at a dilution of 1:300. The cells were incubated for 1 h in secondary antibody and Hoescht dye (NucBlue, Thermo Fisher) before imaging under an inverted fluorescent microscope at 20X.

To obtain cell volume measurements, differentiated cardiomyocytes were passaged onto Matrigel-coated glass bottom dishes at a low concentration. Two different cell batches were used for each genotype. The cells were cultured in RPMI/B27 (including insulin) for 7 days after which they were incubated in 10 μM CellTracker Green CMFDA Dye (Thermo Fisher) and 16.2 μM Hoechst Dye (Life Technologies) diluted in RPMI for 45 min at 37°C. Following a media rinse, the cells were imaged using a Leica confocal microscope at a 40× magnification. Several Z-stacks were recorded for each randomly selected cell and the image sets were analyzed using Imaris (Oxford Instruments). The thresholds, filters and algorithms used during the volume analysis were standardized across both cell groups.

### Western blot

For each cell line, two different batches of cells were collected into a microcentrifuge tube. Cells were sheared using a 26G needle and homogenized in RIPA buffer supplemented with sodium orthovanadate, PMSF, protease inhibitor cocktail, and phosphatase inhibitor cocktail (Santa Cruz Biotechnology). The protein was separated on precast 4–20% Mini-PROTEAN TGXgels (Bio-Rad) before transfer to a PVDF membrane (Millipore). Samples were normalized using a BCA total protein assay kit (Thermo Fisher). A primary antibody solution was prepared using anti-histone H3 in mouse (Abcam) and anti-myosin heavy chain in rabbit (Thermo Fisher). The membrane was incubated in the primary antibody solution overnight at 4°C. The membranes were imaged on a Li-Cor Odyssey scanner. Quantification and analysis were performed using ImageJ.

### Statistical analysis

Results are given as the mean with standard deviation. For a comparison of two groups, statistical significance was determined using Student's t-test with a confidence level of *P* < 0.05. For paired analysis with repeated measurements on the same samples, paired *t* tests were used with a confidence level of *P* < 0.05. For comparison of groups under multiple conditions, two-way and three-way ANOVA was used followed by pairwise comparison using post-hoc testing with Tukey correction to determine significant differences with a confidence level of *P* < 0.05.

## Supplementary Material

pgad011_Supplementary_Data

## Data Availability

All data generated or analyzed during this study are included in this published article and its supplementary information files.
