# Supplementary material for: Mechanisms of pathogenicity in the hypertrophic cardiomyopathy-associated TPM1 variant S215L
Source: PNAS Nexus. 2023 Jan 21;2(3):pgad011. doi: 10.1093/pnasnexus/pgad011 (PMC9991458; doi:10.1093/pnasnexus/pgad011)
Supplement: pgad011_Supplementary_Data [file pgad011_supplementary_data.docx]

**
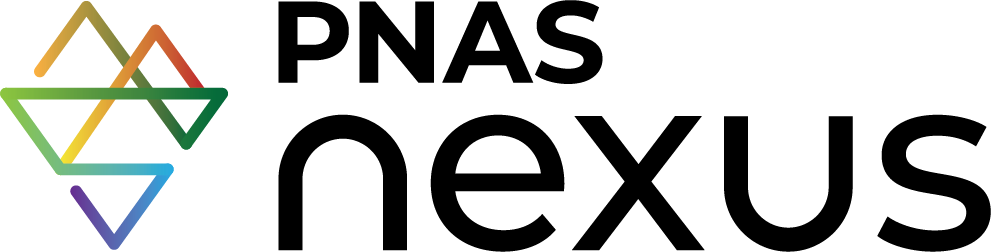
**

**Supplementary Information for**

**Mechanisms of Pathogenicity in the Hypertrophic Cardiomyopathy-Associated TPM1 Variant S215L**

Saiti S Halder^1^, Michael J Rynkiewicz^4^, Jenette G Creso^1^, Lorenzo R Sewanan^1,2^, Lindsey Howland^3^, Jeffrey R Moore^3^, William Lehman^4^, Stuart G Campbell^1^

1. Department of Biomedical Engineering, Yale University
2. Department of Internal Medicine, Columbia University
3. Department of Biological Sciences, University of Massachusetts Lowell
4. Department of Physiology/Biophysics, Boston University

**Corresponding Author:**

Stuart G Campbell
[stuart.campbell@yale.edu](mailto:stuart.campbell@yale.edu)
10 Hillhouse Avenue

PO Box- 208267
New Haven, CT- 06520-8267

**This PDF file includes:**

Supplementary text

Figures S1 to S2

**Investigating changes in cell and tissue architecture due to the impact of a S215L mutation**

**Results**

**No change in cross-sectional areas**

Cross sectional areas were calculated for tissues in both rounds of the experiments outlined in the main manuscript. Sample OCT images are shown in Figure S1A and the cross-sectional areas for Round 1 and Round 2 of the experiments are shown in Figure S1B and S1C respectively. Round 1 and Round 2 refers to the EHTs whose contractile properties are detailed in Figure 4 and Figure 9 respectively. For both rounds of experiments, the cross-sectional area remained consistent and there were no significant differences between the groups.

**No difference in resting sarcomere length**

Tissues were stained for alpha-actinin and imaged using a confocal microscope (Figure S2A). Calculation of mean separation between the Z-disks labelled using alpha-actinin showed no statistical difference between the resting sarcomere lengths (Figure S2B). The mean length ranged between 2.10 μm to 2.18 μm for all three groups.

**Methods**

**Tissue Cross Sectional Areas**

Engineered Heart Tissues (EHTs) undergo remodeling throughout the duration of culture, which may result in altered tissue thickness at the end of the culture period. To correctly assess the tissue thickness, Engineered Heart Tissues (EHT) cross sections were imaged using Optical Coherence Tomography (OCT). The cross-sectional areas of the images were calculated using ImageJ.

**Resting Sarcomere Length Measurement**

EHTs were fixed in 4% paraformaldehyde (PFA) for 15 minutes and stained with alpha-actinin (1:100) and Hoechst. Slides were imaged using a confocal microscope (40x). Multiple images were captured for each EHT for a total of 6 EHTs per group that were seeded in two separate batches. 10 well visible strands were selected for each EHT. The intensities across the strand were plotted and the distance between the peaks were recorded to calculate the mean separation between bands in each strand.

| **** |
| --- |
| **Fig. S1.** Tissue Cross Sectional Area. (A) shows sample OCT images obtained for EHTs from each group. The resulting cross sectional areas calculated using ImageJ for the EHTs in Round 1 (Those corresponding to Figure 4 of the main manuscript) and Round 2 (Those corresponding to Figure 9 of the main manuscript) are shown in (B) and (C) respectively. |

| **** |
| --- |
| **Fig. S2.** Resting Sarcomere Length in EHTs. (A) shows sample images obtained using a confocal microscope at 40x magnification. Red is alpha-actinin and blue is Hoechst nuclear dye. (B) shows the mean resting sarcomere lengths for 60 strands from each group. |
